# Supplementary material for: State transitions in the substantia nigra reticulata predict the onset of motor deficits in models of progressive dopamine depletion in mice
Source: eLife. 2019 Mar 6;8:e42746. doi: 10.7554/eLife.42746 (PMC6402832; doi:10.7554/eLife.42746)
Supplement: Transparent reporting form [file elife-42746-transrepform.docx]

***eLife’s* transparent reporting form**

We encourage authors to provide detailed information *within their submission* to facilitate the interpretation and replication of experiments. Authors can upload supporting documentation to indicate the use of appropriate reporting guidelines for health-related research (see [EQUATOR Network](http://www.equator-network.org/%20)), life science research (see the [BioSharing Information Resource](https://biosharing.org/)), or the [ARRIVE guidelines](http://www.plosbiology.org/article/info:doi/10.1371/journal.pbio.1000412) for reporting work involving animal research. Where applicable, authors should refer to any relevant reporting standards documents in this form.

If you have any questions, please consult our Journal Policies and/or contact us: [editorial@elifesciences.org](mailto:editorial@elifesciences.org).

**Sample-size estimation**

- You should state whether an appropriate sample size was computed when the study was being designed
- You should state the statistical method of sample size computation and any required assumptions
- If no explicit power analysis was used, you should describe how you decided what sample (replicate) size (number) to use

Please outline where this information can be found within the submission (e.g., sections or figure legends), or explain why this information doesn’t apply to your submission:

No explicit power analysis was used. Numbers of animals used for the study were based on standard group sizes established previously in the literature (Wang et al., Brain Research 1324, 2010; Breit et al., J. Neuroscience Res., Jan. 2013; Delaville et al., Neuroscience 202, 2012; Tseng et al., Eur. J. Neurosci. 22, 2005; Tsai et al., FASEB J. 17, 2003; Diaz et al., Mov. Dis. 18, 2003). Numbers of neurons in each group reflect the number of well-isolated single units that could be recovered from recordings in each animal.

**Replicates**

- You should report how often each experiment was performed
- You should include a definition of biological versus technical replication
- The data obtained should be provided and sufficient information should be provided to indicate the number of independent biological and/or technical replicates
- If you encountered any outliers, you should describe how these were handled
- Criteria for exclusion/inclusion of data should be clearly stated
- High-throughput sequence data should be uploaded before submission, with a private link for reviewers provided (these are available from both GEO and ArrayExpress)

Please outline where this information can be found within the submission (e.g., sections or figure legends), or explain why this information doesn’t apply to your submission:

Each mouse was used for a single session of behavioral testing and in vivo recordings.

All mice in which recordings were performed were used for the study unless no TH loss was observed due to poor placement of the infusion cannulas. Criteria used to define well-isolated single units were: (i) PCA clusters were significantly different (P < 0.001); (ii) J3-statistic was greater than 1; (iii) percentage of ISI violations (<2 ms) was less than 0.15%; (iv) Davies-Bouldin test statistic was less than 0.5; (v) under manual verification, optical stimulation did not occlude the ability to delineate single units from noise.

**Statistical reporting**

- Statistical analysis methods should be described and justified
- Raw data should be presented in figures whenever informative to do so (typically when N per group is less than 10)
- For each experiment, you should identify the statistical tests used, exact values of N, definitions of center, methods of multiple test correction, and dispersion and precision measures (e.g., mean, median, SD, SEM, confidence intervals; and, for the major substantive results, a measure of effect size (e.g., Pearson's r, Cohen's d)
- Report exact p-values wherever possible alongside the summary statistics and 95% confidence intervals. These should be reported for all key questions and not only when the p-value is less than 0.05.

Please outline where this information can be found within the submission (e.g., sections or figure legends), or explain why this information doesn’t apply to your submission:

Statistical analysis (this description is provided in the Methods of the article): All data sets were tested for normality with the Shapiro-Wilk test and equal variance with Levene’s test prior to any statistical analysis. Data are expressed as median ± median absolute deviation (MAD) unless otherwise indicated. N values reported in text are formatted as follows: n = # of neurons/# of animals. Statistical analysis regarding firing rate and CVISI was performed using Kruskal-Wallis analysis of variance (ANOVA) nonparametric test (KW) and any differences were further investigated by Kruskal-Wallis pairwise comparison between condition of interest and saline controls with a Bonferroni correction for number of comparisons. Statistical analysis regarding proportion of bursting units across conditions was performed using a Pearson Chi-Square Test (Pearson) and any further differences were investigated by z-test comparison of column proportions (z-test) between condition of interest and saline controls with a Bonferroni correction for number of comparisons. Statistical analysis regarding the average percentage of synchronous pairs was performed using a one-way ANOVA followed by a Dunnett t (2-sided) post hoc test with the exception of the asymmetric condition which was not normally distributed, thus we ran a KW test as described above. Results of initial statistical tests can be found in the figure legends, whereas any post-hoc testing is reported in results text where appropriate. A p-value of 0.05 was considered statistically significant. All statistical procedures were performed using IBM SPSS Statistics, version 24 and MATLAB 2018a.

Raw data: In each graph, data from each individual mouse are shown and n’s are provided in the text. The total number of neurons in each group are reported. As this number is typically in the hundreds, we cannot show individual data for each neuron and instead display the data as a box plot showing median, Q1, Q3, min, and max for each group.

(For large datasets, or papers with a very large number of statistical tests, you may upload a single table file with tests, Ns, etc., with reference to sections in the manuscript.)

**Group allocation**

- Indicate how samples were allocated into experimental groups (in the case of clinical studies, please specify allocation to treatment method); if randomization was used, please also state if restricted randomization was applied
- Indicate if masking was used during group allocation, data collection and/or data analysis

Please outline where this information can be found within the submission (e.g., sections or figure legends), or explain why this information doesn’t apply to your submission:

Samples were allocated into experimental groups based on TH immunoreactivity after data were collected and analyzed. Therefore, analyses were done blind to condition and group allocation was based on a quantitative metric.

**Additional data files (“source data”)**

- We encourage you to upload relevant additional data files, such as numerical data that are represented as a graph in a figure, or as a summary table
- Where provided, these should be in the most useful format, and they can be uploaded as “Source data” files linked to a main figure or table
- Include model definition files including the full list of parameters used
- Include code used for data analysis (e.g., R, MatLab)
- Avoid stating that data files are “available upon request”

Please indicate the figures or tables for which source data files have been provided:

Raw values that went into each graph, as well as accompanying stats and code are available at https://github.com/KidElectric/willard2018a
